# Ustekinumab Persistence in Biologic-Experienced Crohn’s Disease Patients: A Single-Center Retrospective Cohort Study

**DOI:** 10.3390/medicina62071327

**Published:** 2026-07-09

**Authors:** Fatih Eren, Mehmet Refik Goktug, Resul Akduman, Muhammed Abdurrahman Celik, Selim Gurel

**Affiliations:** Division of Gastroenterology, Department of Internal Medicine, Faculty of Medicine, Bursa Uludag University, Bursa 16059, Turkey; mrgoktug@hotmail.com (M.R.G.); resulakduman@uludag.edu.tr (R.A.); drmuhammedcellik@gmail.com (M.A.C.); gurels@uludag.edu.tr (S.G.)

**Keywords:** Crohn’s disease, ustekinumab, treatment persistence, biologic-experienced patients, real-world study

## Abstract

*Background and Objectives*: Ustekinumab has demonstrated high treatment persistence in Crohn’s disease (CD), particularly in biologic-experienced populations. However, the impact of cumulative biologic exposure and disease severity on persistence remains insufficiently characterized in real-world settings. *Methods*: This single-center retrospective cohort study included adult CD patients initiating ustekinumab between 2017 and 2026. All patients had prior biologic exposure. Treatment persistence was evaluated using Kaplan–Meier analysis, and subgroup comparisons were performed using log-rank tests. *Results*: A total of 64 biologic-experienced patients were included (mean age 38.3 ± 13.2 years; 50% female). Treatment persistence was 89.7% at 12 months and 79.1% at 24 months, with a median follow-up of 25.5 months. A total of 13 discontinuation events occurred, most commonly due to lack of efficacy (10.9%), followed by extraintestinal manifestations (4.7%). Disease location and prior surgery were significantly associated with reduced persistence (log-rank *p* = 0.040 and *p* = 0.031, respectively). Log-rank analysis demonstrated significant differences in treatment persistence according to disease location (*p* = 0.040) and prior surgery (*p* = 0.031), while penetrating disease behavior showed a borderline association (*p* = 0.056). *Conclusions*: Ustekinumab demonstrated high treatment persistence in this biologic-experienced Crohn’s disease cohort. Lower treatment persistence appeared to be associated with ileocolonic disease location and prior surgery. Given the limited number of discontinuation events, these subgroup findings should be interpreted cautiously. Nevertheless, our findings support ustekinumab as a durable real-world treatment option for biologic-experienced Crohn’s disease patients.

## 1. Introduction

Crohn’s disease (CD) is a chronic relapsing-remitting disease that can affect any segment of the gastrointestinal tract and is characterized by transmural inflammation. It may exhibit a progressive disease course, leading to complications such as strictures, fistulas, and abscesses [1,2]. Anti-TNF therapies are currently the first-line treatment for patients with moderate-to-severe disease activity or an aggressive disease course. However, approximately 40% of patients do not respond to these therapies, and up to 50% of responders lose their response within the first year [3].

Anti-TNF treatment failure is associated with disease progression and poor clinical outcomes in CD. Primary nonresponse or secondary loss of response may result in increased hospitalization, need for surgical intervention, and development of complications due to ongoing inflammation. In addition, this situation increases the need for switching to alternative biologic agents and makes the treatment algorithm more complex. Patients with anti-TNF failure generally have a more refractory disease phenotype and a less favorable long-term prognosis [4].

Ustekinumab is a fully human monoclonal antibody targeting the interleukin-12/23 complex [5]. Since its approval for the treatment of moderate-to-severe CD, ustekinumab has gained popularity as a biologic therapy in the IBD population because of its favorable efficacy and safety profile [6,7].

Treatment persistence with biologic therapies refers to the duration for which a patient continues the initiated biologic agent without permanent discontinuation. In real-world studies, treatment persistence is considered a clinically meaningful outcome because it reflects multiple factors, including treatment effectiveness, safety, tolerability, patient preference, physician decision-making, and treatment accessibility. However, treatment persistence should not be interpreted as a direct surrogate for clinical or endoscopic remission [8]. Observational studies in Crohn’s disease have consistently demonstrated high treatment persistence with ustekinumab, particularly among biologic-experienced patients, supporting its durability in routine clinical practice [9]. In most real-world cohorts, 1-year persistence rates with ustekinumab have been reported to exceed 80%, and the most common reasons for treatment discontinuation have been secondary loss of response and inadequate clinical response. In addition, ustekinumab’s better tolerability compared with other biologic agents has emerged as an important factor contributing to continued treatment [9,10].

This retrospective cohort study aimed to present real-world data on biologic-experienced patients with CD who initiated ustekinumab treatment at our hospital between 2017 and 2026. In particular, we aimed to analyze treatment persistence rates, treatment discontinuation and its causes, the development of CD-related complications, and the need for surgery.

## 2. Materials and Methods

### 2.1. Study Design and Patient Selection

This single-center, retrospective cohort study included adult patients with a diagnosis of Crohn’s disease who were biologic-experienced and started ustekinumab treatment at our center between January 2017 and March 2026.

### 2.2. Inclusion Criteria

Age ≥ 18 years at the initiation of ustekinumab treatment

Confirmed diagnosis of CD based on clinical, endoscopic, radiologic, and histopathologic findings

Initiation of ustekinumab for the treatment of CD

At least one follow-up record after ustekinumab treatment

Treatment and follow-up performed at the center where the study was conducted

Having received at least one dose of ustekinumab

### 2.3. Exclusion Criteria

Patients under 18 years of age

Non-CD diagnoses (e.g., infectious colitis, undiagnosed colitides)

Patients who had used ustekinumab before presenting to the study center and whose baseline data could not be obtained

Patients with missing essential clinical data required for analysis

Patients with no follow-up data after ustekinumab treatment

Patients enrolled in interventional clinical trials involving ustekinumab

Patients who discontinued treatment for nonmedical reasons (excluded from analysis when necessary)

### 2.4. Data Collection

Demographic, clinical, laboratory, and treatment-related data were retrospectively obtained from electronic medical records. Demographic data included age, sex, height, weight, body mass index (BMI), smoking status (never, current, former), disease duration, Montreal classification (age, location, behavior), presence of perianal disease, prior conventional treatments (methotrexate, 5-ASA, azathioprine, corticosteroids) and biologic treatments (infliximab, adalimumab, certolizumab pegol, vedolizumab), extraintestinal manifestations (EIMs), immune-mediated inflammatory diseases (IMIDs), and comorbidities, as well as surgical history before and after ustekinumab, reason for discontinuation of ustekinumab treatment, adverse events, and laboratory parameters such as white blood cell count (WBC), neutrophil count, hemoglobin, platelet count, C-reactive protein (CRP), erythrocyte sedimentation rate (ESR), albumin, and ferritin.

### 2.5. Treatment Protocol

Ustekinumab treatment was administered in all patients according to the standard dosing regimen. Induction therapy was given intravenously as weight-based dosing (260 mg for ≤55 kg, 390 mg for 56–85 kg, and 520 mg for >85 kg). Following induction, all patients received maintenance therapy with 90 mg of subcutaneous ustekinumab every 8 weeks. No dose intensification strategy, including interval shortening or intravenous re-induction, was performed before treatment discontinuation in this cohort.

### 2.6. Follow-Up

Patients were followed at our inflammatory bowel disease outpatient clinic according to routine clinical practice. Following ustekinumab induction, patients were generally evaluated every 8–12 weeks during maintenance therapy. Additional visits were scheduled when clinically indicated because of disease activity, adverse events, or treatment-related concerns. At follow-up visits, treatment continuation status, adverse events, need for surgery, and available laboratory parameters were assessed and recorded in the electronic medical record system. Because this was a retrospective real-world cohort study, the exact number of follow-up visits varied among patients according to clinical needs and disease activity and was therefore not analyzed as a study outcome.

### 2.7. Definitions

Treatment persistence was defined as the time from initiation of ustekinumab treatment to discontinuation and was expressed in months. Temporary interruptions in ustekinumab administration were not considered treatment discontinuation if treatment was subsequently resumed. No patients were lost to follow-up during the study period.

All permanent ustekinumab discontinuations, including discontinuation due to death or unknown reasons, were considered treatment discontinuation events in the Kaplan–Meier analysis.

Biologic experience was defined as prior use of at least one biologic agent before ustekinumab.

CD-related surgery was defined as surgical interventions performed because of disease complications developing after ustekinumab treatment.

### 2.8. Endpoints

The primary endpoint of the study was ustekinumab treatment persistence, which was selected as the principal real-world outcome measure. Owing to the retrospective design and the lack of standardized longitudinal assessments, clinical remission, biochemical remission, and endoscopic remission were not included as predefined study endpoints. Secondary endpoints were reasons for treatment discontinuation, need for surgery, adverse events, and factors associated with treatment persistence.

### 2.9. Statistical Analysis

Continuous variables were expressed as mean ± standard deviation (SD) or median and interquartile range (IQR), as appropriate. Categorical variables were presented as frequencies and percentages. Treatment persistence was estimated using the Kaplan–Meier method, and differences between subgroups were assessed using the log-rank test. Given the limited number of treatment discontinuation events, no Cox proportional hazards regression analysis was performed. Statistical analyses were conducted using IBM SPSS Statistics for Windows, Version 28.0 (IBM Corp., Armonk, NY, USA). A two-sided *p* value < 0.05 was considered statistically significant.

## 3. Results

### 3.1. Baseline Demographic and Clinical Characteristics

A total of 64 patients were included in the study, comprising 32 men (50%) and 32 women (50%). The mean age of the patients was 38.3 ± 13.2 years, and the median age was 36.5 years (IQR: 26.8–49.0). The mean body mass index (BMI) was 22.0 ± 4.0 kg/m^2^. According to smoking status, 40.6% of the patients had never smoked, 26.6% were current smokers, 10.9% were former smokers, and smoking history was unknown in 21.9%.

According to the Montreal classification, the majority of patients were diagnosed between 17 and 40 years of age (A2: 75.0%). A total of 17.2% of the patients were in the A1 group, and 7.8% were in the A3 group. The most common disease location was ileocolonic involvement (L3: 50.0%), followed by ileal involvement (L1: 37.5%) and colonic involvement (L2: 12.5%). Regarding disease behavior, penetrating disease (B3) was the most common phenotype (43.8%). Non-stricturing, non-penetrating disease (B1) was observed in 31.3% of patients, whereas stricturing disease (B2) was observed in 25.0%. Perianal disease was present in 15.6% of the patients.

All patients were biologic-experienced. While 57.8% of the patients had previously used one biologic agent, 31.3% had received two biologic agents, and 10.9% had received three or more biologic therapies. When conventional therapies before ustekinumab were examined, 84.4% of the patients had used azathioprine, 64.1% had used 5-aminosalicylic acid (5-ASA), and 57.8% had used corticosteroids. Methotrexate use was very low (1.6%). Among previous advanced therapies, 73.4% of the patients had used infliximab, 53.1% adalimumab, and 21.9% vedolizumab. Certolizumab use was present in a smaller number of patients (4.7%), as was upadacitinib use (7.8%).

The mean leukocyte count was 8.6 ± 3.2 × 10^9^/L, and the mean neutrophil count was 5.6 ± 2.8 × 10^9^/L. The mean hemoglobin level was 12.2 ± 2.2 g/dL, and the mean platelet count was 346.6 ± 143.0 × 10^9^/L. Regarding inflammatory markers, the median CRP level was 16.6 mg/L (IQR 5.9–55.8), the median ESR was 20.0 mm/h (IQR 11.0–43.0), and the median ferritin level was 71.0 ng/mL (IQR 19.8–110.0). The mean albumin level was 40.4 ± 6.4 g/L. The baseline demographic and clinical characteristics of the study population are summarized in Table 1.

### 3.2. Treatment Status and Reasons for Ustekinumab Discontinuation

During follow-up, a high rate of treatment persistence with ustekinumab was observed. Kaplan–Meier analysis demonstrated a treatment persistence rate of 79.1% at 24 months. Separately, 51 of 64 patients (79.7%) were receiving ongoing ustekinumab treatment at the time of data collection. Among patients who discontinued treatment, the most common reason was loss of efficacy or inadequate clinical response, accounting for 10.9% of cases.

At a lower frequency, some treatment discontinuations were related to extraintestinal manifestations. Reasons in this group included sacroiliitis, pyoderma gangrenosum, or switching to a JAK inhibitor for rheumatologic indications, and these occurred in a total of 4.7% of the patients.

One patient (1.6%) died during the study. This patient had undergone surgery because of perianal drainage and subsequently died from multiple organ failure due to intra-abdominal sepsis. Treatment status and reasons for ustekinumab discontinuation are presented in Table 2.

### 3.3. Surgical History and Types of Prior Surgery

Twenty-nine patients (45.3%) had a previous surgical history. The most common surgical procedures included ileocolic resection and fistula surgeries. In addition, ostomy creation was observed in 4 patients (6.3%), and colonic resections were observed in 2 patients (3.1%). During follow-up, only 3 patients (4.7%) required surgery after ustekinumab treatment, and no additional surgery was performed in the vast majority of patients (95.3%). Findings related to prior surgeries are presented in Table 3.

### 3.4. Ustekinumab Treatment Persistence (Kaplan–Meier Analysis)

**Figure 1:**
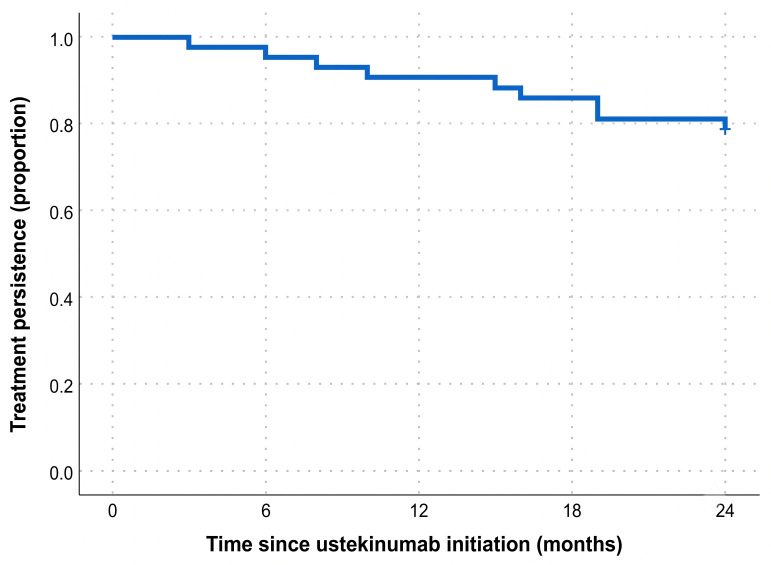
Kaplan–Meier curve showing treatment persistence following ustekinumab initiation. Tick marks indicate censored observations.

Ustekinumab treatment persistence was evaluated using Kaplan–Meier analysis. The treatment continuation rate was 89.7% at month 12 and 79.1% at month 24. The median follow-up duration was 25.5 months. Inspection of the Kaplan–Meier curve showed a low rate of early treatment discontinuation and a gradual decline over time. Treatment persistence for the entire cohort is shown in Figure 1.

Subgroup Kaplan–Meier analyses were performed according to biologic exposure, prior surgery, extraintestinal manifestations, disease location, disease behavior, and age groups. Visual inspection of the survival curves suggested differences in treatment persistence across several clinical subgroups. Formal comparisons were subsequently performed using log-rank tests.

Kaplan–Meier curves and log-rank analyses showed that treatment persistence could differ significantly across some clinical subgroups. In the analysis by disease location, a significant difference was found between groups (log-rank *p* = 0.040), and lower persistence was observed particularly in patients with ileocolonic involvement (L3). In terms of disease behavior, patients with a penetrating (B3) phenotype showed a tendency toward lower persistence, although this difference did not reach statistical significance (log-rank *p* = 0.056).

**Figure 2:**
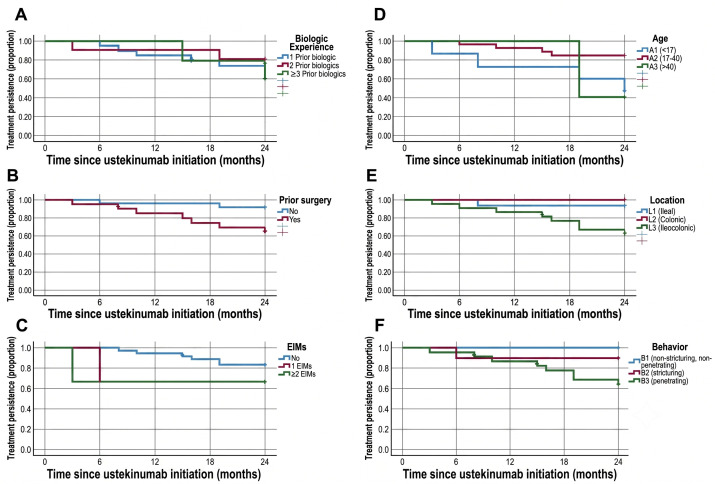
Kaplan–Meier curves of ustekinumab treatment persistence according to clinical subgroups. (**A**) Biologic experience, (**B**) prior surgery, (**C**) extraintestinal manifestations (EIMs), (**D**) age groups, (**E**) disease location, and (**F**) disease behavior. Differences between groups were assessed using the log-rank test. A statistically significant difference was observed for disease location (*p* = 0.040) and prior surgery (*p* = 0.031), while other comparisons did not reach statistical significance (all *p* > 0.05). **Given the limited number of events, subgroup analyses should be interpreted with caution.**

Prior surgical history had a significant effect on treatment persistence, and lower persistence was observed in patients with a surgical history (log-rank *p* = 0.031). No significant difference in treatment persistence was found between age groups (log-rank *p* = 0.126). Kaplan–Meier curves for subgroup analyses are shown in Figure 2.

### 3.5. Safety and Adverse Events

No treatment-related adverse events were identified in the available medical records during the study period. No infusion or injection reactions, malignancies, or serious adverse events attributed to ustekinumab were recorded. One patient died during follow-up because of multiple organ failure secondary to intra-abdominal sepsis following surgery for perianal disease. This event was considered related to disease complications and postoperative sepsis rather than ustekinumab treatment.

## 4. Discussion

Treatment persistence has emerged as an important real-world outcome in Crohn’s disease because it reflects treatment continuation under routine clinical practice and is influenced by multiple factors, including treatment effectiveness, safety, tolerability, physician decision-making, patient preference, and treatment accessibility. Therefore, treatment persistence should not be interpreted as a direct surrogate for clinical response, clinical remission, biochemical remission, or endoscopic healing. The primary objective of the present study was to evaluate real-world treatment persistence and safety of ustekinumab in biologic-experienced patients rather than objective treatment efficacy. Due to the retrospective design, standardized longitudinal assessments of disease activity indices (e.g., CDAI or Harvey–Bradshaw Index), biomarkers, and endoscopic outcomes were not consistently available and therefore could not be analyzed systematically. Nevertheless, treatment persistence remains a clinically meaningful endpoint that has been widely used in observational studies evaluating the long-term performance of biologic therapies in Crohn’s disease.

In the study by Bressler et al. [10] evaluating 8724 patients with CD, 12- and 24-month persistence rates were 82.9% and 71.4%, respectively. In their subgroup analysis based on biologic experience, treatment persistence was significantly higher in biologic-naïve patients with CD (HR: 0.72; CI: [0.65–0.79]) [10]. In a meta-analysis including 903 biologic-experienced patients with CD, 12-month persistence for ustekinumab was 80.1%; however, the number of biologics previously used and the reasons for treatment discontinuation were not examined [11]. In a prospective study evaluating highly refractory CD that was unresponsive to both anti-TNF agents and vedolizumab, 12-month persistence was reported as 78.1%, and the most important reason for treatment discontinuation was lack of response [6]. In another study including 57 patients with stricturing Crohn’s disease, the 12- and 24-month persistence rates were 78% and 76%, respectively. Increased bowel wall thickness on imaging, prior biologic use, and the duration of obstructive symptoms were factors associated with treatment discontinuation [12]. Increasing biologic exposure may reflect a more refractory disease phenotype, which is associated with reduced treatment persistence. In the present study, persistence rates at 12 and 24 months were similar and nonresponse to treatment was an important reason for discontinuation.

Similar findings have been reported in the SUSTAIN study, a large real-world cohort evaluating the long-term effectiveness and safety of ustekinumab in Crohn’s disease. The study demonstrated sustained effectiveness and an acceptable safety profile in a predominantly biologic-experienced population. Although our study evaluated treatment persistence rather than standardized measures of treatment efficacy, the high persistence observed in our cohort is consistent with the long-term treatment continuation reported in the SUSTAIN study [13].

Previous real-world studies have demonstrated high treatment persistence with ustekinumab in Crohn’s disease, particularly in biologic-experienced populations. However, these studies have largely classified patients in a binary manner as biologic-naïve or biologic-experienced, without accounting for the cumulative burden of prior biologic exposure. In contrast, the present study provides a more granular assessment by stratifying patients according to the number of prior biologic therapies.

The high treatment persistence observed in our study among patients with CD and multiple biologic exposures may also have been influenced by health system-related factors. In Türkiye, due to the stepwise biologic treatment algorithm, anti-TNF agents must be used as first-line therapy. In addition, difficulties in obtaining vedolizumab at our center during the COVID-19 pandemic may have limited treatment options. Furthermore, upadacitinib and other JAK inhibitors were approved only in the later period of the study and were not accessible early on for most patients. For these reasons, the limited availability of alternative treatment options may have increased the tendency of both patients and clinicians to continue ustekinumab therapy, thereby contributing to the high persistence rates observed. This effect may have been more pronounced in our study given the high proportion of patients with a history of multiple biologic therapies. The therapeutic landscape of Crohn’s disease continues to evolve with the introduction of newer biologic agents and selective cytokine inhibitors. Notably, the SEQUENCE trial demonstrated that risankizumab was noninferior to ustekinumab for clinical remission and superior for endoscopic remission in patients with prior anti-TNF exposure [14]. These findings may influence future treatment sequencing strategies in biologic-experienced Crohn’s disease patients. Nevertheless, ustekinumab remains an important therapeutic option with well-established long-term real-world effectiveness, safety, and persistence data [13,14].

Extraintestinal manifestations appear to play an important role in treatment decisions in Crohn’s disease. Previous studies have shown that ustekinumab may have limited efficacy, particularly in conditions such as axial spondyloarthritis and sacroiliitis, and this may necessitate a change in therapy [15]. The ASAS-EULAR recommendations consider ustekinumab ineffective or only minimally effective in axial spondyloarthritis/sacroiliitis [16]. Similarly, a systematic review published in 2021 reported that it did not demonstrate efficacy in IBD, particularly in axial spondyloarthritis [17]. In a retrospective cohort of patients with concomitant IBD and ankylosing spondylitis/spondyloarthritis, treatment was modified in 85% of patients at the time of the second diagnosis; initiation, switching, or augmentation of biologic therapy was associated with clinical improvement [18]. In addition, a post hoc analysis of the UNITI trials reported that ustekinumab did not provide a significant improvement compared with placebo in the overall burden of extraintestinal manifestations at 6 and 52 weeks of follow-up in CD [19].

By contrast, in dermatologic extraintestinal manifestations, especially pyoderma gangrenosum, there are reports of favorable responses. IL-23-mediated inflammation and neutrophil activation, which are involved in the pathogenesis of pyoderma gangrenosum, share immunologic mechanisms with Crohn’s disease and psoriasis [20]. In several studies evaluating patients with IBD and extraintestinal manifestations, ustekinumab has shown high response rates in psoriasis, pyoderma gangrenosum, and erythema nodosum [17,21]. Although ustekinumab has been reported to achieve high response rates in dermatologic extraintestinal manifestations, treatment responses may be heterogeneous, and inadequate control of these manifestations in some patients may lead to discontinuation of therapy. In the present study, extraintestinal manifestations were among the reasons for treatment discontinuation in a substantial number of patients.

Ustekinumab is considered a promising option for the prevention of postoperative recurrence (POR) or for the treatment of established POR in CD. In a multicenter study published in 2021, ustekinumab was reported to be more effective than azathioprine in preventing endoscopic POR [22]. The 2023 ENEIDA registry study also suggested that ustekinumab and vedolizumab appear to be effective in preventing POR in high-risk patients [23]. In a recent study evaluating POR, among 44 patients with a Rutgeerts score ≥ i2, ustekinumab achieved endoscopic success in 50.0% and clinical success in 72.7% of cases [24]. In contrast, anti-TNF agents are generally preferred as the first-line option for prevention of POR in the literature. In one study, although the sample size was limited, anti-TNF therapy was found to be more effective than ustekinumab and vedolizumab when initiated within the first four weeks after ileocecal resection in CD [25]. It may be used as an alternative in patients who are unresponsive or unsuitable for anti-TNF therapy. In the present study, the decline in persistence among postoperative patients may have been related to switching to anti-TNF agents.

These findings further support the role of ustekinumab as a durable treatment option in biologic-experienced Crohn’s disease in routine clinical practice. The high treatment persistence observed in our cohort suggests sustained treatment continuation in this difficult-to-treat population. However, given the retrospective design and the lack of standardized clinical, biochemical, and endoscopic outcome assessments, treatment persistence should not be interpreted as direct evidence of long-term treatment efficacy.

No treatment-related adverse events were documented in our cohort. Although the retrospective design may have resulted in underreporting of minor adverse events, no serious adverse events attributable to ustekinumab were identified during follow-up. The single death observed in the study occurred in the setting of postoperative intra-abdominal sepsis and was considered related to disease complications rather than ustekinumab treatment.

This study has several limitations. First, its retrospective design may have introduced selection and information bias. Second, the single-center setting and relatively small sample size, together with the limited number of treatment discontinuation events, may have reduced the statistical power of subgroup analyses and limited the generalizability of our findings. Third, although treatment persistence represents a clinically meaningful real-world outcome, it should not be considered a direct surrogate for treatment efficacy. Because standardized longitudinal assessments of clinical disease activity (e.g., CDAI or Harvey–Bradshaw Index), biochemical markers, and endoscopic outcomes were not routinely available at predefined follow-up intervals, objective measures of treatment response and remission could not be systematically evaluated. Finally, the retrospective nature of the study precluded standardized follow-up schedules, and patient assessments were performed according to routine clinical practice rather than a predefined study protocol. Therefore, our findings should be interpreted as reflecting real-world treatment persistence rather than objective treatment efficacy.

## 5. Conclusions

Ustekinumab demonstrated high treatment persistence in this biologic-experienced Crohn’s disease cohort. Lower treatment persistence appeared to be associated with ileocolonic disease location and prior surgery. Given the limited number of discontinuation events, these subgroup findings should be interpreted cautiously. Overall, our findings support the durability of ustekinumab treatment in routine clinical practice among biologic-experienced patients, while acknowledging that treatment persistence should not be interpreted as a direct measure of treatment efficacy.

## Figures and Tables

**Table 1:** Baseline demographic and clinical characteristics of the study population.

Parameter	Value (*n* = 64)
**Age (years)**	38.3 ± 13.2
**Disease duration (years)**	8.75 ± 10.05
**Sex,** ***n*** **(%)**	
Female	32 (50.0%)
Male	32 (50.0%)
**BMI (kg/m^2^)**	22.0 ± 4.0
**Smoking status,** ***n*** **(%)**	
Non-smoker	26 (40.6%)
Current smoker	17 (26.6%)
Ex-smoker	7 (10.9%)
Unknown	14 (21.9%)
**Montreal classification,** ***n*** **(%)**	
**Age at diagnosis**	
A1 (<17 years)	11 (17.2%)
A2 (17–40 years)	48 (75.0%)
A3 (>40 years)	5 (7.8%)
**Location**	
L1 (ileal)	24 (37.5%)
L2 (colonic)	8 (12.5%)
L3 (ileocolonic)	32 (50.0%)
L4 (isolated upper disease)	0 (0%)
**Behavior**	
B1 (non-stricturing, non-penetrating)	20 (31.3%)
B2 (stricturing)	16 (25.0%)
B3 (penetrating)	28 (43.8%)
**Perianal disease,** ***n*** **(%)**	10 (15.6%)
**Conventional therapies prior to ustekinumab,** ***n*** **(%)**	
Corticosteroids	37 (57.8%)
5-aminosalicylic acid (5-ASA)	41 (64.1%)
Azathioprine	54 (84.4%)
Methotrexate	1 (1.6%)
**Advanced therapies prior to ustekinumab,** ***n*** **(%)**	
Infliximab	47 (73.4%)
Adalimumab	34 (53.1%)
Certolizumab pegol	3 (4.7%)
Vedolizumab	14 (21.9%)
Upadacitinib	5 (7.8%)
**Biologic experience,** ***n*** **(%)**	
1 prior biologic	37 (57.8%)
2 prior biologics	20 (31.3%)
≥3 prior biologics	7 (10.9%)
**Laboratory parameters**	
WBC (×10^9^/L)	8.6 ± 3.2
Neutrophils (×10^9^/L)	5.6 ± 2.8
Hemoglobin (g/dL)	12.2 ± 2.2
Platelets (×10^9^/L)	346.6 ± 143.0
CRP (mg/L)	16.6 (5.9–55.8)
ESR (mm/h)	20.0 (11.0–43.0)
Albumin (g/L)	40.4 ± 6.4
Ferritin (ng/mL)	71.0 (19.8–110.0)

Data are presented as mean ± SD or median (IQR) for continuous variables and as *n* (%) for categorical variables.

**Table 2:** Treatment status and reasons for treatment discontinuation.

Treatment Status	*n* (%)
Ongoing treatment Discontinued treatment **Reasons for discontinuation (*****n*** **= 13)**	51 (79.7) 13 (20.3)
Active disease/loss of response	7 (53.8)
Extraintestinal manifestations	3 (23.1)
Death	1 (7.7)
Unknown	2 (15.4)

**Table 3:** Previous surgical history before ustekinumab treatment.

Parameter	*n* (%)
No prior surgery, *n* (%)	35 (54.7)
Prior surgery, *n* (%)	29 (45.3)
Type of prior surgery, *n* (%)	
Ileocolic resection (including right hemicolectomy)	14 (21.9)
Left hemicolectomy	1 (1.6)
Segmental bowel resection	1 (1.6)
Colon resection	2 (3.1)
Ostomy (ileostomy/colostomy)	4 (6.3)
Fistula-related surgery (fistulotomy/fistulectomy/seton)	7 (10.9)
Appendectomy *	2 (3.1)
Other (enterovesical fistula excision)	1 (1.6)

* Surgery categories were not mutually exclusive. Some patients underwent more than one surgical procedure before ustekinumab initiation. Prior surgery included abdominal and perianal procedures performed before ustekinumab treatment, including appendectomy in patients initially evaluated for suspected Crohn’s disease.

## Data Availability

The datasets used and/or analyzed during the current study are available from the corresponding author upon reasonable request.

## Abbreviations

BMI	Body mass index
CD	Crohn’s disease
CRP	C-reactive protein
EIMs	Extraintestinal manifestations
ESR	Erythrocyte sedimentation rate
IMIDs	Immune-mediated inflammatory diseases
POR	Postoperative recurrence
WBC	White blood cell count
